# Predicting molecular initiating events using chemical target annotations and gene expression

**DOI:** 10.1186/s13040-022-00292-z

**Published:** 2022-03-04

**Authors:** Joseph L. Bundy, Richard Judson, Antony J. Williams, Chris Grulke, Imran Shah, Logan J. Everett

**Affiliations:** 1 Biomolecular and Computational Toxicology Division, Center for Computational Toxicology and Exposure, Office of Research and Development, U.S. Environmental Protection Agency, 109 T.W. Alexander Drive, Durham, NC 27709 USA; 2 Chemical Characterization and Exposure Division, Center for Computational Toxicology and Exposure, Office of Research and Development, U.S. Environmental Protection Agency, 109 T.W. Alexander Drive, Durham, NC 27709 USA

**Keywords:** Molecular initiating events, Chemical safety screening, Machine learning, High throughput transcriptomics, Binary classification, RefChemDB, Library of integrated cellular signatures

## Abstract

**Background:**

The advent of high-throughput transcriptomic screening technologies has resulted in a wealth of publicly available gene expression data associated with chemical treatments. From a regulatory perspective, data sets that cover a large chemical space and contain reference chemicals offer utility for the prediction of molecular initiating events associated with chemical exposure. Here, we integrate data from a large compendium of transcriptomic responses to chemical exposure with a comprehensive database of chemical-protein associations to train binary classifiers that predict mechanism(s) of action from transcriptomic responses. First, we linked reference chemicals present in the LINCS L1000 gene expression data collection to chemical identifiers in RefChemDB, a database of chemical-protein interactions. Next, we trained binary classifiers on MCF7 human breast cancer cell line derived gene expression profiles and chemical-protein labels using six classification algorithms to identify optimal analysis parameters. To validate classifier accuracy, we used holdout data sets, training-excluded reference chemicals, and empirical significance testing of null models derived from permuted chemical-protein associations. To identify classifiers that have variable predicting performance across training data derived from different cellular contexts, we trained a separate set of binary classifiers on the PC3 human prostate cancer cell line.

**Results:**

We trained classifiers using expression data associated with chemical treatments linked to 51 molecular initiating events. This analysis identified and validated 9 high-performing classifiers with empirical *p*-values lower than 0.05 and internal accuracies ranging from 0.73 to 0.94 and holdout accuracies of 0.68 to 0.92. High-ranking predictions for training-excluded reference chemicals demonstrating that predictive accuracy extends beyond the set of chemicals used in classifier training. To explore differences in classifier performance as a function of training data cellular context, MCF7-trained classifier accuracies were compared to classifiers trained on the PC3 gene expression data for the same molecular initiating events.

**Conclusions:**

This methodology can offer insight in prioritizing candidate perturbagens of interest for targeted screens. This approach can also help guide the selection of relevant cellular contexts for screening classes of candidate perturbagens using cell line specific model performance.

**Supplementary Information:**

The online version contains supplementary material available at 10.1186/s13040-022-00292-z.

## Background

Animal-based chemical screening methods have evaluated only a fraction of commercial chemicals for human safety [1]. New approach methodologies (NAMs) can increase the pace of chemical safety testing by using high-throughput cell-based and in vitro approaches capable of rapidly screening thousands of chemicals simultaneously [2]. Establishing alternatives to traditional animal testing is a high priority for the US Environmental Protection Agency (EPA), and a challenge that can be met by developing a multidisciplinary, tiered-testing approach [3]. The success of this strategy relies on the development of methods for rapidly evaluating hazards across diverse and large chemical inventories. One technology that offers utility for this goal is high-throughput transcriptomics (HTTr). Several HTTr platforms have been used to explore chemical bioactivity by interrogating the transcriptomic consequences of chemical exposure in cell lines [4, 5].

One challenge of leveraging HTTr for chemical screening is the analysis and interpretation of large data sets in which hundreds or thousands of gene expression levels are measured across many chemicals and concentrations. Interpretation of such datasets requires time-intensive and specific application of expert knowledge or, more favorably, a systematic analysis that discounts noise and indicates the probability of “true” biological signals that are relevant to chemical hazard identification. One common strategy for distilling this wealth of expression information is to use gene set enrichment analysis to identify molecular pathways perturbed by chemical treatment [6]. However, while this approach may identify molecular networks perturbed by chemical exposure, it may fail to identify the protein(s) on which a chemical initially acts. The Adverse Outcome Pathway (AOP) framework provides a useful context for the distinction between these initial drug-protein interactions, and downstream molecular consequences [7]. In this paradigm, chemicals interact with biological systems through a series of events, beginning with a direct interaction with a biomolecule, such as a protein. This first step of an AOP is commonly called a Molecular Initiating Event (MIE). MIEs in turn cause downstream key events at the molecular, cellular, and organ level, ultimately culminating in an adverse outcome, such as disease, impaired development, or impaired reproduction. Predicting the induction of MIEs from downstream transcriptional consequences of chemical exposure is a formidable challenge in identifying chemical hazards.

Machine learning (ML) methods provide a flexible framework to address this problem. To date, ML methods have leveraged chemical structure and gene expression data to predict chemical targets for pharmaceutical repositioning [8–14], and to predict drug induced liver injury [15]. One such study demonstrated that ML-based classifiers trained on gene expression data can successfully predict therapeutic use categories for candidate pharmaceuticals [13]. Other ML-based methods leverage a variety of data types using a similarity approach to identify likely chemical-protein interactions by training a single multiclassification model [14]. While these previous studies demonstrate the utility of ML approaches to predicting chemical activity from gene expression information, they have limitations. The prediction of chemical activity at the resolution of use categories lacks the gene level granularity of MIE-level predictions [13]. Additionally, many ML-based investigations have used gene expression profiles derived from multiple cell lines in classifier training, thus complicating the identification of cell line specific responses to chemical treatment [13, 14]. In this context, there is value in developing models that account for cell line dependent effects in predicting chemical activity from gene expression at the resolution of MIEs. As an early component of a tiered chemical testing strategy, MIE predictions from HTTr data may offer insight into hazard prioritization and assay selection for potency estimation.

**Fig. 1:**
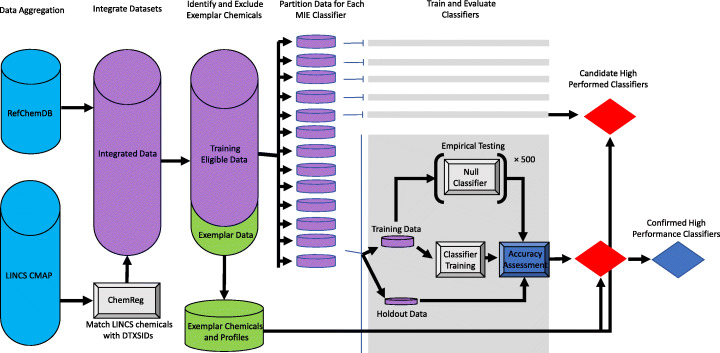
Diagram of data processing and classifier training procedure. From left to right, LINCS chemical identifiers are matched to DTXSIDs using ChemReg, then combined with chemical-target annotations from RefChemDB to produce the integrated data for model training and evaluation. A subset of “exemplar” chemicals that are associated with MIEs to be modeled are excluded from all training data sets for validation purposes. Training data sets for each MIE classifier are then partitioned, and classifiers are trained with 5-fold cross validation using the *caret* package in R. 500 “null” classifiers are generated simultaneously for the purpose of empirical significance testing. Performance for each classifier is evaluated using a MIE-specific holdout data set, internal accuracy, and empirical significance testing, identifying a set of candidate high performance classifiers. This set of candidate high performance classifiers undergoes a final phase of screening using exemplar chemical-based validation

Here, we predict MIEs through a ML-based classification approach (Fig. 1). We developed a methodology for predicting MIEs in specific cell lines by combining data from RefChemDB [16], a database of chemical-target interactions compiled from multiple sources, and LINCS [17], a compendium of in vitro gene expression profiles associated with chemical and genetic perturbagens of specific cell lines. Reasoning that some MIEs may not be well predicted in certain cell types, we utilized a binary classification approach (as opposed to a multiclassification approach), where separate binary classifiers were trained for each MIE, thus allowing classifiers showing poor performance to be easily excluded from the analysis framework.

Classifiers were trained using one of six algorithms: 1) support vector machine with linear kernel (SVM_L), 2) support vector machine with radial kernel (SVM_R), 3) support vector machine with polynomial kernel (SVM_P), 4) K-nearest neighbor (KNN), 5) multilayer perceptron with multiple hidden layers (MLP), and 6) Naïve Bayes (NB). We tested several sets of input features for model training, including pathway scoring. Binary classifiers were validated by four complementary approaches: 1) 5-fold cross validation, 2) validation on holdout data, 3) empirical significance testing, and 4) evaluation of exemplar chemical activity predictions. Lastly, in the interest of testing whether MIE prediction from transcriptomic profiles is dependent on cell line, we compared the predictive accuracy of classifiers trained on data from the MCF7 breast cancer derived cell line versus classifiers trained on data from the PC3 prostate cancer derived cell line.

## Materials and methods

To predict MIEs from gene expression, we trained binary classifiers using chemical-target labels from RefChemDB [16] and gene expression profiles from the LINCS L1000 gene expression compendium [17]. All data analysis was done in the R statistical framework using version 3.6.0.

### RefChemDB chemical-MIE annotations

To train binary classifiers to predict MIEs from gene expression, we leveraged annotations from RefChemDB [16], a database of interactions between chemicals and proteins distilled via automated curation from 15 different resources, including ChEMBL [18], the Comparative Toxicogenomics Database [19], and Drugbank [20]. We downloaded supplemental materials published in [16] containing 339,008 chemical protein annotations. MIE annotations were derived by making minor modifications to RefChemDB entries. Records where the “mode” of interaction between a chemical and protein was “unspecified” were excluded to avoid incorporating chemicals that may have opposing modes of action into the same model. Next, we generated a “MIE” label for each RefChemDB entry by concatenating the RefChemDB “target” and “mode” fields. Finally, we resolved conflicting records that had the same chemical and protein but opposing modes by retaining the record with the highest “support level” (the number of unique literature sources annotated in RefChemDB that support the interaction).

Some MIEs in RefChemDB are annotated for similar or identical sets of chemicals. For example, if filtered to include only annotations with a support level of at least 5, there are 83 chemicals linked to Carbonic anhydrase 1 (CA1) and Carbonic anhydrase 2 (CA2) inhibition, 82 of which are shared. This was expected, as members of the same gene family that have considerable homology (60% in the case of CA1 and CA2 [21]) often have high affinity for a common set of ligands. On the other hand, there are well documented examples of ligands that display variable binding affinity for receptors that share considerable sequence homology [22]. However, resolving MIE activation at the resolution of individual paralogs of a gene family is untenable because there are so few chemicals annotated as uniquely interacting with specific paralogs in most cases, and these are insufficient to train classifiers for any MIEs. Instead, we developed a data-driven strategy for combining MIEs with similar sets of associated chemicals. We first generated a metric of similarity between every pair of MIEs annotated in RefChemDB by calculating the Jaccard index based on the proportion of associated chemicals that are shared by each pair $\left(J\left(A,B\right)=\frac{\left|A\bigcap B\right|}{\left|B\bigcup A\right|}\right)$. This similarity matrix was then converted into dissimilarity by subtracting every value from 1. Similar MIEs were clustered into groups based on their relatedness using the ‘hclust’ R function. Finally, we used the ‘cutree’ R function with h = 0.7 to group similar MIEs together for model training, and manually generated names for the resulting clusters (Supplemental File 1).

### LINCS L1000 gene expression data

To train classifiers to predict MIEs using gene expression, we required a gene expression data set to train models that had sufficient overlap with chemicals annotated in RefChemDB. To meet this need, we used the LINCS L1000 gene expression database [17]. This compendium contains gene expression profiles derived from data collections spanning 83 cell lines exposed to over 20,000 chemicals. The number of chemicals and gene expression profiles is variable across cell types represented in LINCS L1000 data (Supplemental Fig. 1), with the largest number of profiles generated in MCF7 (42,049) and PC3 (35,154) cells, derived from human breast cancer and prostate cancer respectively. Therefore, we initially trained classifiers using only profiles generated from MCF7 cells and subsequently trained corresponding classifiers using only PC3-derived profiles.

Level 5 data (moderated Z-scores) were downloaded from the gene expression omnibus [23] for LINCS phase1 and phase2. The LINCS metadata use a customized primary identifier for chemical perturbagens (Broad_IDs) that have not been harmonized or mapped to other chemical databases. To match LINCS perturbagens with chemicals annotated in RefChemDB, LINCS chemicals were registered into ChemReg and curated using processes described previously [24]. Registering chemicals into ChemReg produces unique DSSTox Substance Identifiers (DTXSIDs) that are publicly available on the CompTox Chemicals Dashboard [https://jcheminf.biomedcentral.com/articles/10.1186/s13321-017-0247-6] (https://comptox.epa.gov/dashboard/), a web-based application providing access to chemical property data and in vivo and in vitro toxicity data for nearly 900,000 chemicals. Broad_IDs were matched to DTXSIDs in ChemReg using additional chemical descriptors extracted from LINCS metadata files, including canonical SMILES, InChIKeys, and chemical names (Supplemental File 2). 4,284 of 21,299 (20%) unique chemical identifiers in LINCS were successfully mapped to a DTXSID using ChemReg.

Most chemicals present in the LINCS data set have multiple associated gene expression profiles. Some chemicals were screened at multiple concentrations and with varying durations, resulting in multiple profiles for a single chemical, in addition to replicate experiments generating profiles with identical conditions. The number of profiles associated with each chemical varies from chemical to chemical, with a median of 6 profiles in MCF7 cells for the LINCS chemicals mapped to a DTXSID in RefChemDB. Previous ML-based investigations have limited the heterogeneity of training data by restricting training data sets to a single concentration and exposure duration [25]. We sought to leverage the multiple gene expression profiles available for each chemical to increase the available data for classifier training, and therefore did not restrict gene expression profiles based on treatment duration or concentration. However, a subset of chemicals represented in LINCS (90 compounds) were associated with over 20 gene expression profiles and thus have the potential to dominate training data sets. To limit the amount of information that any one chemical can contribute to training data sets, a maximum of 20 gene expression profiles (selected at random) per chemical were used in classifier training. Finally, gene expression measurements in the form of moderated Z-scores were standardized by subtracting from every probe that probe’s mean across all gene expression profiles and dividing by the standard deviation using the ‘preProcess’ function in R library *caret* with “center” and “scale” methods, as done in previous ML-based investigations using LINCS data [10] .

### Binary classification algorithms

To optimize models, we evaluated the performance of six different classification algorithms for model training. A subset of these algorithms were based on Support Vector Machines (SVMs). SVMs are a popular class of algorithm for biological binary classification problems [26]. SVMs trained with linear, polynomial, and radial basis kernels are denoted as SVM_L, SVM_P, and SVM_R, respectively. Multi-layer perceptrons have been shown previously to outperform SVM-based classification approaches in predicting chemical use categories from L1000 gene expression data [13]. To see if this held true for our analysis, we also trained models using Multi-layer Perceptrons (MLP), as well as two other popular classification algorithms: Naïve Bayes (NB) and k-nearest neighbor (KNN). All classifier training was done using the *caret* package v6.0–83 in the R statistical framework [27]. SVM, MLP, and NB classifiers were trained using calls to the *kernlab* v0.9–27, *RSNNS v0.4-12*, and *naivebayes* v0.9.6 R libraries, respectively. Hyperparameter tuning for model optimization was performed automatically by *caret* using the grid search default in the ‘train_control’ function.

### Input features

Previous ML-based investigations using LINCS L1000 data have shown that pre-processing gene-level expression information into pathway scores can significantly improve multi-classifier performance [13]. We therefore tested a set of classifiers generated using pathway features. Pathway scores were calculated using a modified version of single-sample Gene Set Enrichment Analysis (ssGSEA), as described previously [28, 29] using all LINCS L1000 genes (landmark + inferred genes) for 2232 pathways derived from the canonical pathways set of MSigDB (v7.0) [30]. Pathway scores were then normalized using the identical procedure as gene-level standardization.

To explore how the selection of input features affects classifier performance, we trained models using three types of L1000 derived features: 1) Moderated Z-scores for all genes (LINCS landmark and inferred), 2) Moderated Z-scores for LINCS landmark genes only, and 3) Pathway scores.

### Model training and evaluation approach

The classifier training and validation pipeline is outlined in Fig. 1. Briefly, our approach was to train a binary classifier for each MIE by labeling LINCS L1000 gene expression profiles with MIE labels based on annotations in RefChemDB. We focused on RefChemDB annotations with a support level of at least 5, as previous analyses demonstrated relatively high agreement between RefChemDB annotations and ToxCast assays at or above this support level [16]. First, we identified suitable MIEs for modeling using two criteria: 1) MIEs must be linked to at least 5 chemicals in RefChemDB by at least 5 literature sources each (support level > = 5) and; 2) the total number of LINCS profiles associated with the set of MIE-linked chemicals in the target cell line must be ≥50. LINCS perturbagens (and their associated gene expression profiles) lacking high-confidence annotation in RefChemDB (support level > = 5) were excluded from model training. Using these criteria, we identified 51 MIEs with sufficient data for modeling.

**Fig. 2:**
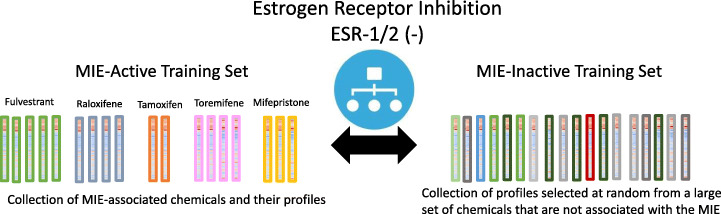
Example schematic of training data set structure for ESR-1/2 (−) classifier. Binary classifiers were trained using “MIE-Active” and “MIE-Inactive” collections of gene expression profiles that are equal in number. In this example, the MIE-Active set is comprised of profiles derived from chemical treatments that are associated with ESR1 or ESR2 inhibition based on RefChemDB annotations. The MIE-Inactive training set is comprised of an equal number of profiles derived from chemical treatments that have no annotation for ESR1 or ESR2 in RefChemDB

For each MIE, training data sets were formed using RefChemDB chemical-MIE linkages to identify and label suitable LINCS gene expression profiles. First, a collection of “MIE-active” LINCS profiles was generated by selecting LINCS profiles associated with chemicals that are linked to a given MIE. Then, an equally sized collection of “MIE-inactive” profiles was generated by randomly selecting profiles that met two criteria: 1) profiles must not be associated with chemical treatments linked to the MIE being modeled at any support level nor with any mode and 2) profiles must be associated with a different MIE with a support level ≥ 5 (Fig. 2). After aggregating equally sized MIE-active and MIE-inactive profiles, 20% of profiles in each set were randomly selected as holdout data and excluded from model tuning and training. Binary classifiers were trained on the remaining profiles with 5-fold cross validation and hyperparameter selection using the *caret* package in R [27]. This process is summarized in pseudocode below.

- For each MIE with sufficient training data:
  - Generate list of “MIE-active” chemicals associated with MIE from RefChemDB
  - Generate collection of *N* MIE-active profiles linked to MIE-active chemical treatments in the selected cell line
  - Generate collection of *N* MIE-inactive profiles linked to chemicals that are not annotated for the MIE
  - Select 20% each of MIE-active and MIE-inactive profiles as holdout data
  - Remaining 80% of profiles are designated as training data set
  - For each algorithm in (SVM_L, SVM_P, SVM_R, NB, KNN, MLP)
  - For each unique combination of hyperparameters (grid search)
    - Train classifier using 5-fold cross validation:
      - Split model training data into 5 equal subsets
      - For each subset *k*:
        - Combine non-*k* subsets and train model on these data
          - Test model on *k*
- Identify optimal hyperparameter configuration that returns the highest average accuracy (proportion of correct assignments) across the 5 folds
  - Test accuracy of model with optimal hyperparameters using holdout data
  - Return internal (average accuracy from 5-fold cross validation) and holdout accuracies for optimal hyperparameter configuration

### Assessment of model performance

Classifier training and holdout data sets were comprised of equal numbers of MIE-Active and MIE-Inactive profiles (Fig. 2). Because these data sets are balanced with respect to labels, we used accuracy, defined here as the proportion of correct assignments $\left(\frac{TP+ TN}{TP+ TN+ FP+ FN}\right)$ as an intuitive metric for measuring classifier performance. Internal accuracy for each model was taken as the mean accuracy score from 5-fold cross validation as calculated by *caret* and extracted from the results object returned by the ‘train’ function. For hyperparameter tuning, the hyperparameter configuration and resulting trained model association with the highest internal accuracy was used for all further analysis. To account for possible overfitting, we used holdout data to calculate holdout accuracy independent of training data. However, this approach faces a challenge due to 1:many relationships between chemicals and profiles in LINCS. Gene expression profiles in the holdout data set were selected at random from the pool of MIE-active and MIE-inactive profiles in which there are often multiple profiles for any one chemical treatment. Thus, while the individual profiles in holdout data are distinct from those used for training, there is overlap in which chemical treatments were performed for profiles in the training and holdout data sets. This may introduce unwanted bias in measuring classifier accuracy with the holdout data set. Previous ML studies focused on predicting drug induced liver injury from LINCS data have addressed this challenge by partitioning all profiles associated with a given chemical into either training or holdout data sets [15]. Unfortunately, this solution was not suitable for the current study, as most MIEs have too few associated chemicals to partition training and validation data on a per-chemical basis. To address this challenge in the current study, we supplemented our evaluation on holdout data with an additional collection of training-excluded exemplar chemicals and an empirical significance assessment.

### Model validation with empirical significance testing

Previous ML-based analyses of LINCS data restricted training data to one profile per perturbagen [5]. In the interest of training robust classifiers by leveraging the wealth of gene expression profiles in LINCS, we opted to include up to 20 profiles per chemical in model training. However, we recognized that this approach might be biased towards MIEs with a small set of annotated chemicals. Such classifiers might achieve spuriously high accuracies simply due to the relatedness of profiles derived from the same chemical treatment in the MIE-active set, without identifying mechanism of action signatures shared across MIE-active chemicals. To compensate for this possible source of bias, we adapted an empirical significance methodology used previously in investigations of quantitative trait loci, and in ML-based study of drug induced liver injury [15, 31].

We ascertained the value of information added for MIE-active labels by generating null classifiers for each MIE. To generate training data sets for null models, training data were randomized by swapping each chemical in the MIE-active set with a single replacement chemical, selected at random from the set of chemicals with at least as many available profiles in LINCS. Only chemicals that were associated with at least one other MIE in RefChemDB, but not associated with the original classifier’s MIE, were eligible to be selected as replacement chemicals. Thus, null classifiers were trained on profiles derived from the same number of chemicals as their “original” counterparts, but with no association to the target MIE. After randomly selecting chemicals to be used in training, the number of LINCS profiles to be used in classifier training for each replaced chemical was limited to the number of profiles associated with the MIE-active chemical from the original model. Thus, null models were trained on the same number of LINCS profiles, distributed across the same number of chemicals as the original model from which they were derived.

Null classifiers followed the same training and validation procedures as original models. Internal and holdout validation accuracy were recorded for each iteration of null model training. For each of the 306 classifiers (51 MIEs × 6 algorithms), 500 null data sets were generated and corresponding classifiers trained. Then, an empirical significance test was performed by calculating *p*-values from the proportion of null classifiers that achieved internal accuracies higher than their original counterparts, similar to the strategy used previously [31]. Candidate high-performance models were identified as classifiers that achieved an internal accuracy ≥95% of null model accuracies, corresponding to an empirical *p*-value ≤0.05.

### Classifier validation with exemplar chemicals

To assess model performance independently of internal and holdout accuracies, we excluded a set of “exemplar” chemicals (and all their associated LINCS gene expression profiles) from all training and holdout data sets, including for null classifiers. To select exemplar chemicals, we used a data-driven approach. First, we generated a list of candidate chemicals by taking the union of the top 10 chemicals for each MIE ranked by support level. We then iteratively selected one exemplar chemical for each MIE that did not yet have an exemplar selected, with the constraint that the exclusion of the chemical could not reduce the remaining training data to < 5 chemicals or < 50 total profiles for any MIE on the list. If a selected exemplar chemical was in the list of top 10 supported chemicals for multiple MIEs, that exemplar chemical was assigned to all these MIEs, and all were removed from the list for further exemplar selection. This process identified 31 exemplar chemicals assigned to 44 of the 51 MIEs. All profiles associated with these exemplar chemicals were subsequently excluded from all classifier training (Table 1). For 7 MIEs (51 total MIEs – 44 MIEs with candidate exemplars), no chemical among the candidate exemplars could be excluded from classifier training without resulting in the loss of sufficient training data, so no exemplar chemical was assigned.

**Table 1:** Summary of Available Training Data for MCF7-Trained Classifiers

MIE Name	MIE Active Profiles	MIE Active Chemicals	Exemplar Chemical Name	Exemplar Chemical DTXSID	Exemplar Chemical Support Level
ABCB1 (−)	283	25	Quinidine	DTXSID4023549	20
ACE (−)	57	6	Fosinopril	DTXSID1023079	6
A/B-CHE (−)	50	7	NA	NA	NA
ADRA1A (+)	67	10	Epinephrine	DTXSID5022986	10
ADRA2A (+)	58	7	Epinephrine	DTXSID5022986	8
ADRB-1/2 (−)	67	8	Nadolol	DTXSID3023342	5
ADRB-1/3 (+)	51	6	Epinephrine	DTXSID5022986	8
ADRB2 (+)	55	9	Epinephrine	DTXSID5022986	5
ALOX5 (−)	51	5	MK 886	DTXSID5041067	5
APP (−)	71	5	NA	NA	NA
AR (−)	56	5	Progesterone	DTXSID3022370	7
AR (+)	52	8	17-Methyltestosterone	DTXSID1033664	10
CA-9/12 (−)	76	10	4-(2-Aminoethyl)benzenesulfonamide	DTXSID20188814	10
CA-many (−)	51	7	NA	NA	NA
CA-1/2 (−)	90	13	(RS)-(+/−)-sulpiride	DTXSID1042574	36
CYP19A1 (−)	70	7	Naringenin	DTXSID1022392	10
CYP2D6 (−)	52	5	Quinidine	DTXSID4023549	34
CYP3A4 (−)	144	13	Quinidine	DTXSID4023549	6
DHFR/TYMS (−)	56	6	Methotrexate	DTXSID4020822	43
DRD2 (−)	118	14	Haloperidol	DTXSID4034150	50
DRD2 (+)	125	15	Dopamine	DTXSID6022420	26
EGFR/ERBB2 (−)	140	9	Erlotinib	DTXSID8046454	55
ESR-1/2 (−)	68	5	Tamoxifen	DTXSID1034187	30
ESR-1/2 (+)	145	12	17beta-Estradiol	DTXSID0020573	142
FLT1/KDR (−)	122	10	Erlotinib	DTXSID8046454	10
FLT3 (−)	84	5	NA	NA	NA
HDAC (−)	174	10	MS-275	DTXSID0041068	37
HMGCR (−)	50	5	Mevastatin	DTXSID4040684	14
HRH1 (−)	110	14	Astemizole	DTXSID9020110	5
HTR2A (−)	67	8	Haloperidol	DTXSID4034150	11
JAK2 (−)	54	5	NA	NA	NA
KCNH2 (−)	369	34	Haloperidol	DTXSID4034150	14
KIT (−)	88	5	NA	NA	NA
MAO-A/B (−)	75	11	Tranylcypromine	DTXSID2023694	10
MAPK14 (−)	78	5	NA	NA	NA
MET (−)	114	7	Cabozantinib	DTXSID10233968	10
MTOR/PI3K (−)	204	12	Everolimus	DTXSID0040599	5
NR1I2 (+)	50	6	17beta-Estradiol	DTXSID0020573	5
NR3C1 (+)	100	10	Clocortolone pivalate	DTXSID0045460	5
PDE3A (−)	67	5	Cilostamide	DTXSID3045140	5
PDE4-A/B/D (−)	56	5	Piclamilast	DTXSID3040227	9
PDGFRB (−)	66	6	Erlotinib	DTXSID8046454	9
PPAR-A/D/G (+)	137	16	Ciglitizone	DTXSID0040757	38
PTGS-1/2 (−)	247	28	Flurbiprofen	DTXSID0037231	14
SCN-1/2-A (−)	51	5	Lidocaine	DTXSID1045166	5
SCN5A (−)	100	9	Lidocaine	DTXSID1045166	6
SLC22A-1/2 (−)	63	7	Quinidine	DTXSID4023549	14
SLC22A6 (−)	55	6	Methotrexate	DTXSID4020822	6
SLC6A-2/3/4 (−)	192	18	Nisoxetine	DTXSID0045175	11
TOP2A (−)	75	7	Doxorubicin	DTXSID8021480	12
TUB (−)	104	8	Vinblastine	DTXSID8021430	12

### Classifier validation with Kolmogorov–Smirnov test

To further validate classifiers independent of training data, MIE predictions were generated for each classifier for all 76,843 MCF7 derived LINCS profiles. Predictions associated with non-chemical treatments or chemical treatments present in a classifier’s training data set were then excluded. Classifier performance was then assessed using chemicals associated with each MIE in RefChemDB, but at support levels below our initial threshold for training data (i.e. support level = 3 or 4). A subset of associations at this support level are likely spurious [16], however the legitimate associations at this support level should lead to an enrichment of higher prediction scores within this subset of chemicals as compared to other chemicals in the L1000 data with no association to the MIE. When sufficient low-support chemicals are available for a candidate high performance classifier, this enrichment should be separable from random chance when the scores are considered in aggregate. To test this hypothesis, we used a one-tailed Kolmogorov–Smirnov (KS) test. Using the ‘ks.test’ function [32] in R to determine if ranked predictions associated with moderate-support chemicals (combined with a training-excluded exemplar chemical, if available) are significantly higher than the distribution of predictions across all LINCS profiles.

### Comparison of model performance by cellular context

In the context of transcriptomic differences between cell lines at baseline, we hypothesized that some MIE classifiers would perform better using data from some cell lines relative to others. To identify MIEs that show cell type specific differences in prediction, we trained a separate set of models using LINCS gene expression profiles derived from the PC3 prostate cancer derived cell line. PC3 cells differ from MCF7 cells in terms of baseline gene expression and tissue of origin, and thus exhibit a different repertoire of proteins that can be perturbed by chemical treatment. Also, the PC3 cell line has the second highest number of associated gene expression profiles in the LINCS data set (Supplemental Fig. 1), thus ensuring the maximal number of classifiers can be trained for comparison to MCF7-trained counterparts.

## Results

LINCS chemical perturbagens were matched to DTXSIDs which are generated upon chemical registration into the ChemReg chemical registration system [24]. Of 21,299 unique chemicals in LINCS metadata, 4920 (23%) were successfully linked to a DTXSID. LINCS perturbagens were then matched to RefChemDB chemical-MIE annotations. Filtering RefChemDB with a minimum support level of 5 and eliminating conflicting annotations yielded 601 distinct MIEs and 1181 unique chemicals, 765 (65%) of which were represented in LINCS. After integrating RefChemDB and LINCS L1000 meta data, we identified 51 MIEs with sufficient data (≥5 chemicals; ≥50 profiles) for classifier training. We explored 6 algorithms and 3 input feature types, generating a total of 918 distinct classifiers. Binary classifiers were trained for each MIE using balanced sets of “MIE-active” profiles, associated with chemical treatments linked to the MIE through RefChemDB, and a set of “MIE-inactive” profiles, selected at random from chemical treatments with no RefChemDB linkage with the MIE.

### Landmark gene-based models out-perform other feature types

**Fig. 3:**
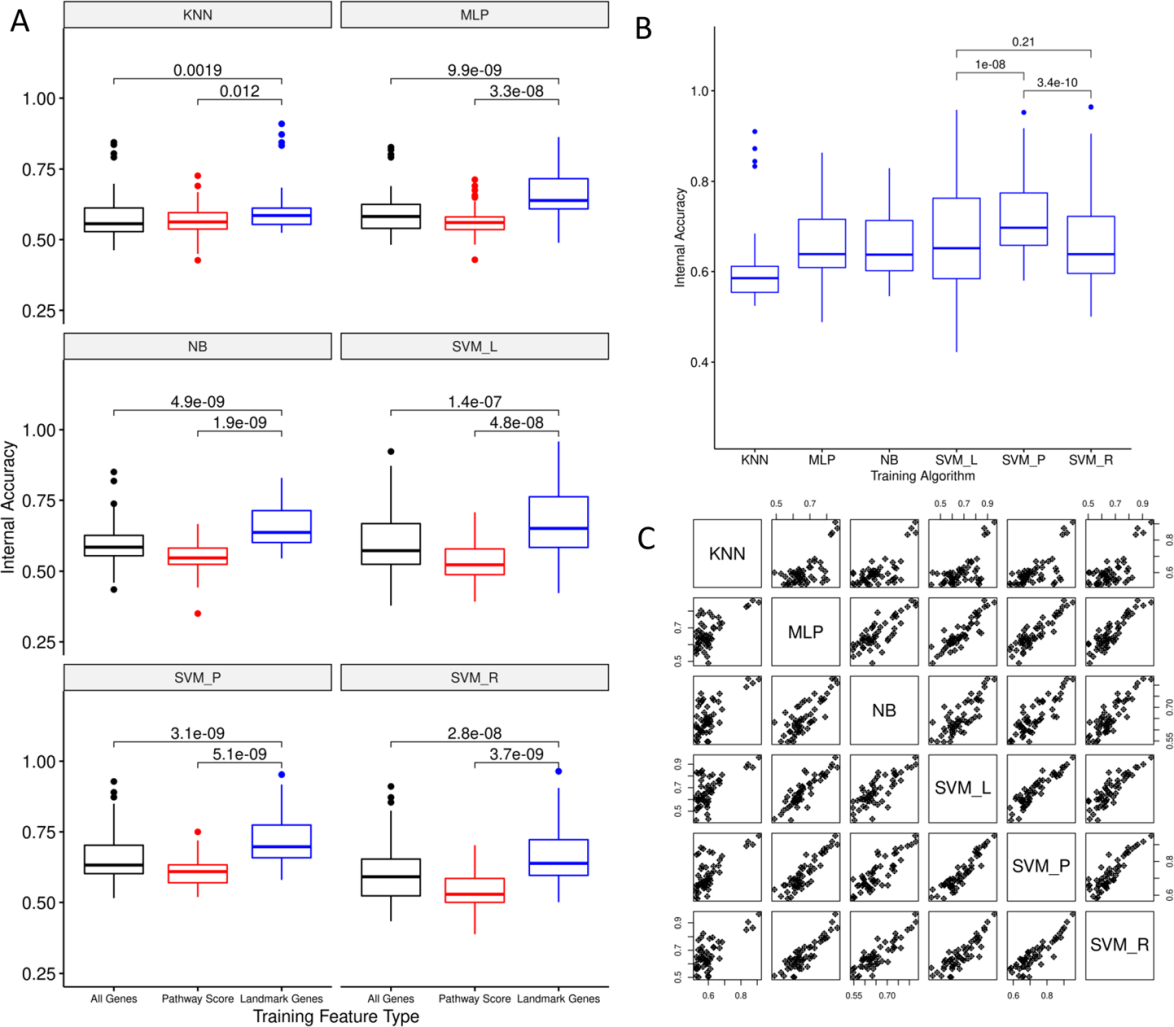
Comparison of internal accuracy scores across input feature types and training algorithms. A) Distributions of internal accuracies for 51 MCF7-trained MIE classifiers are shown as box-and-whisker plots for each combination of 6 classification algorithms and 3 input feature types. Each box spans the inter-quartile range, with the thicker line indicating the median. Whiskers on each plot indicate the range of data within 1.5x IQR beyond the upper and lower quartiles, and outlier data points beyond this range are plotted as individual dots. Within each classification algorithm, paired Wilcoxon tests were performed to determine if accuracies were significantly different when trained on different feature types. *P*-values are denoted for the relationships indicated. B) Distributions of internal accuracies for 51 MCF7-trained landmark gene MIE classifiers are shown as box-and-whisker plots for each classification algorithm. Pairwise Wilcoxon tests were performed to compare the top three performing algorithms (SVM_P, SVM_L, and SVM_R), with *p*-values denoted for the relationships indicated. C) Scatter plots comparing internal accuracies across 6 classification algorithms for MCF7-trained landmark gene classifiers

Classifiers were trained using LINCS L1000 gene expression data in the form of moderated z-scores (LINCS level 5 data). These values are derived from 978 measurements of landmark genes directly measured by the L1000 assay, and inferred expression of an additional 11,350 genes [17]. To determine if the use of inferred genes results in improved classifier accuracy, we first trained classifiers using both landmark and inferred genes. For comparison, we trained a second set of classifiers using landmark genes only. Landmark gene-based classifiers achieved significantly higher internal accuracies than models trained on all LINCS genes, regardless of the classification algorithm used (Fig. 3A, paired Wilcoxon test *p* < 1.9 × 10^− 3^).

Previous ML investigations of LINCS data have shown improved performance for models trained on gene expression data that are pre-processed into pathway scores [13]. To test if this finding holds for our use case, we trained a third set of classifiers on pathway scores derived using ssGSEA [28] on all landmark and inferred genes for 2232 gene sets from the MSigDB canonical pathways collection. Landmark gene classifiers out-performed pathway score classifiers (Fig. 3A, paired Wilcoxon test *p* < 1.2 × 10^− 2^). 79% of landmark gene classifiers out-performed corresponding classifiers using all LINCS genes and pathway scores for the same MIE and classification algorithm. Due to the magnitude of difference in accuracies between feature types, and the consistency of these findings across classification algorithms, we focused on landmark gene-based classifiers for further analysis.

### Optimal training algorithms are MIE-dependent

Focusing on classifiers trained with landmark genes, we then compared performance across training algorithms (Fig. 3B and C). Using the internal accuracy as a metric of algorithm performance, mean accuracies across MIEs ranged from 0.60 for KNN classifiers to 0.73 for SVM_P classifiers. SVM_P classifiers performed significantly better than SVM_L and SVM_R (paired Wilcoxon test, *p*-value = 10^− 8^), which were the algorithms with the next highest mean accuracies of 0.67 and 0.66 respectively.

**Fig. 4:**
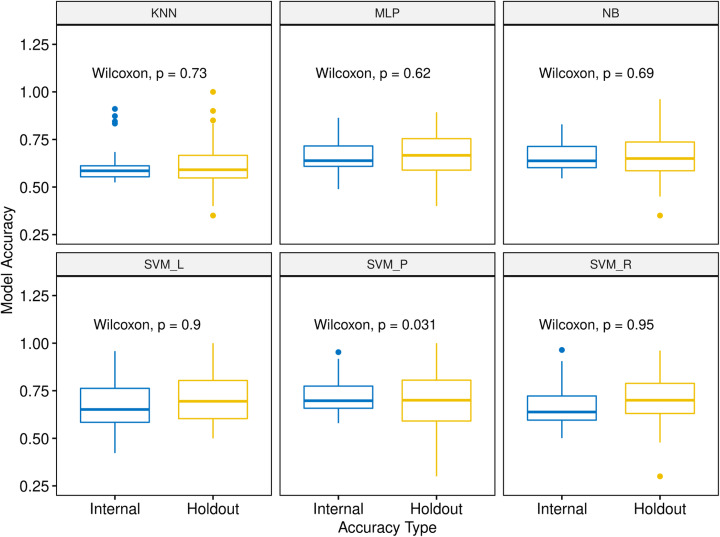
Comparison of internal and holdout accuracy. Distributions of internal and hold-out accuracies for 51 MCF7-trained MIE classifiers are shown as box-and-whisker plots for each classification algorithm. Within each classifier, a paired one-tailed Wilcoxon test was performed to test whether holdout accuracies were greater than internal accuracies across all MIEs

A potential pitfall of ML-based models is overfitting, in which models perform well on training data, but fail to generate accurate predictions on new data. To detect overfitting in models trained by each algorithm, we compared internal accuracies with holdout accuracies using one tailed, paired, Wilcoxon tests (Fig. 4). Internal accuracies were not significantly higher than holdout accuracies with the exception of classifiers trained with SVM_P. Classifiers trained with SVM_P showed significantly lower holdout accuracies than internal accuracies (paired one-tailed Wilcoxon test *p*-value = 0.031), suggesting overfitting. Therefore, classifiers trained using the SVM_P algorithm were excluded from further analysis. In the absence of SVM_P models, there was not a clear winner among ML algorithms with respect to internal accuracies (SVM_L vs SVM_R, paired one-tailed Wilcoxon test *p*-value = 0.21, Fig. 3B). Thus, rather than restricting the analysis to a set of classifiers trained by any one algorithm, we instead considered classifiers from all five remaining algorithms (excluding SVM_P) for further optimization.

### Empirical significance testing identifies high performance classifier candidates

One caveat of the current study design is that MIE classifiers were trained on sets of gene expression profiles, where multiple profiles may correspond to the same chemical treatment. This was necessary to build training data sets with adequate numbers of examples of MIE-linked gene expression changes. However, for MIEs linked to a small set of chemicals, we considered the possibility that the resulting classifiers might achieve spuriously high accuracies simply due to the relative homogeneity of profiles in the MIE-active sets derived from few unique chemical treatments (possibly as few as 5 unique chemicals), without capturing a mechanism of action relevant to the MIE. To explore the possibility, we first compared internal accuracy with the number of chemicals used in classifier training (Supplemental Figure 2A). This analysis revealed a weak trend that MIEs with fewer training chemicals tend to have higher internal accuracies on average. This correlation did not appear statistically significant in most cases, but was consistent across all six training algorithms. We note that some of the most accurate classifiers are trained on the smallest number of chemicals, e.g. Topoisomerase 2-alpha (TOP2A (−)) was trained on 75 profiles corresponding to 7 chemicals and achieved an internal accuracy of 0.88 and an external accuracy of 0.87. Therefore, to more thoroughly assess each classifier for potential issues with training data homogeneity, we opted to use an empirical significance testing approach.

A series of empirical significance tests were performed. The goal of these tests was to ascertain if a classifier’s performance was driven by real gene expression changes associated with the MIE of interest, or, if performance is driven by spurious commonality in gene expression that occurs by chance among any set of *n* random chemicals. For each classifier, a corresponding collection of 500 null classifiers was trained by the random replacement of MIE-active chemicals in the training data set. We then once again evaluated the relationship between training chemical number and spuriously high model accuracy by comparing the mean internal accuracy from each set of 500 null classifiers with the number of chemicals used in the MIE-active set (Supplemental Figure 2B). Mean null accuracies showed a significant and negative correlation with training chemical number, confirming the need for empirical assessments of classifier performance that incorporate null accuracies.

**Fig. 5:**
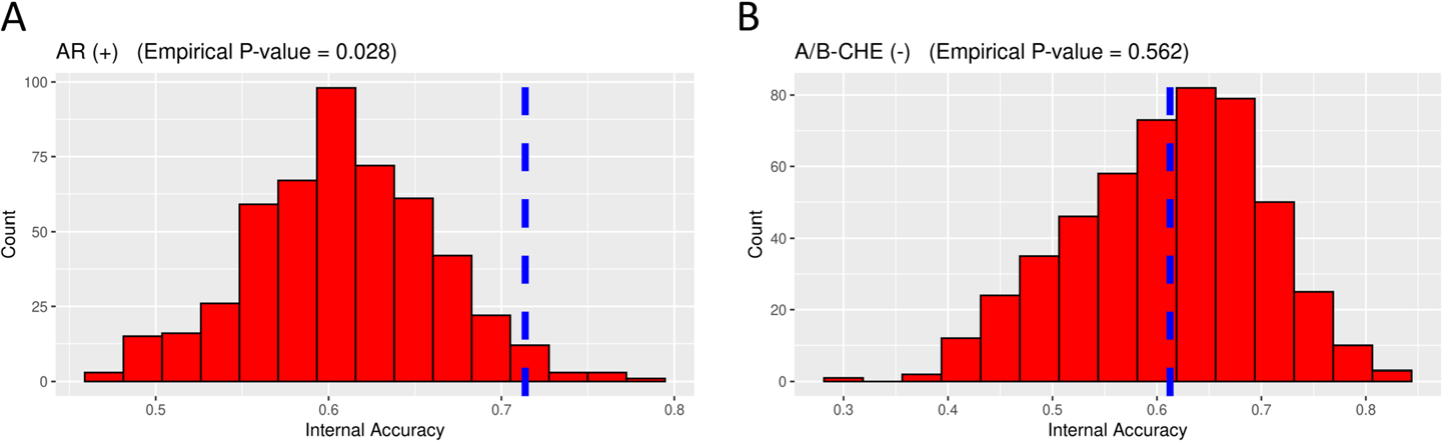
Example of empirical significance tests. Distributions of internal accuracies from 500 null models are shown as histograms. Internal accuracy from the corresponding original model is marked by the blue vertical line. A) Empirical testing results for the AR (+) classifier, which generated an internal accuracy higher than 97.2% of null models, resulting in empirical *p*-value = 0.028. B) Empirical testing results for the A/B-CHE (−) classifier, which generated an internal accuracy score higher than only 43.8% of null models, resulting in empirical *p*-value = 0.562

To estimate the potential of each individual MIE classifier to achieve spurious predictions, we compared the internal accuracy of each original model to the distribution of internal accuracies achieved by corresponding null models using an empirical significance test (Fig. 5). Empirical *p*-values were derived from the proportion of null models that achieved an internal accuracy greater than or equal to that of the original classifier. Classifiers were considered as candidate high performance classifiers if ≤5% of null models out-competed their original counterpart, corresponding to a *p*-value of less than 0.05. For MIEs with multiple classifier algorithms passing this threshold, we selected the algorithm with the highest internal accuracy score. Using these criteria, we identified candidate high performance classifiers for 20 MIEs to focus on for further validation (Table 2). Internal accuracies for these classifiers ranged from 0.65 to 0.94, with a median of 0.77.

**Table 2:** Performance Statistics for MCF7-Trained Candidate High Performance Classifiers

MIE Name	Classification Algorithm	Internal Accuracy	Holdout Accuracy	MIE Active Profiles	MIE Active Chemicals	Mean Null Accuracy	Empirical *p*-value
ADRA2A (+)	SVM_R	0.72	0.86	58	7	0.60	0.03
ALOX5 (−)	NB	0.73	0.55	51	5	0.61	0.01
AR (+)	NB	0.71	0.60	52	8	0.61	0.03
DRD2 (−)	SVM_R	0.68	0.54	118	14	0.58	0.03
ESR-1/2 (−)	MLP	0.89	0.92	68	5	0.69	0.00
ESR-1/2 (+)	SVM_L	0.85	0.79	145	12	0.64	0.00
FLT1/KDR (−)	MLP	0.75	0.69	122	10	0.66	0.02
HDAC (−)	SVM_L	0.82	0.78	174	10	0.67	0.00
HMGCR (−)	MLP	0.79	0.85	50	4	0.66	0.03
HRH1 (−)	MLP	0.71	0.61	110	14	0.61	0.01
JAK2 (−)	SVM_L	0.88	0.85	54	5	0.71	0.01
KCNH2 (−)	SVM_R	0.66	0.64	369	34	0.58	0.00
MAPK14 (−)	SVM_L	0.86	0.93	78	5	0.73	0.03
MET (−)	SVM_L	0.83	0.70	114	7	0.70	0.01
MTOR/PI3K (−)	SVM_R	0.90	0.88	204	12	0.70	0.00
NR3C1 (+)	SVM_R	0.73	0.68	100	10	0.60	0.01
PTGS-1/2 (−)	SVM_R	0.65	0.65	247	28	0.58	0.00
SLC22A6 (−)	KNN	0.70	0.64	55	6	0.58	0.02
TOP2A (−)	SVM_L	0.88	0.87	75	7	0.67	0.00
TUB (−)	SVM_L	0.94	0.90	104	8	0.59	0.00

### Classifier validation with exemplar reference chemicals

**Fig. 6:**
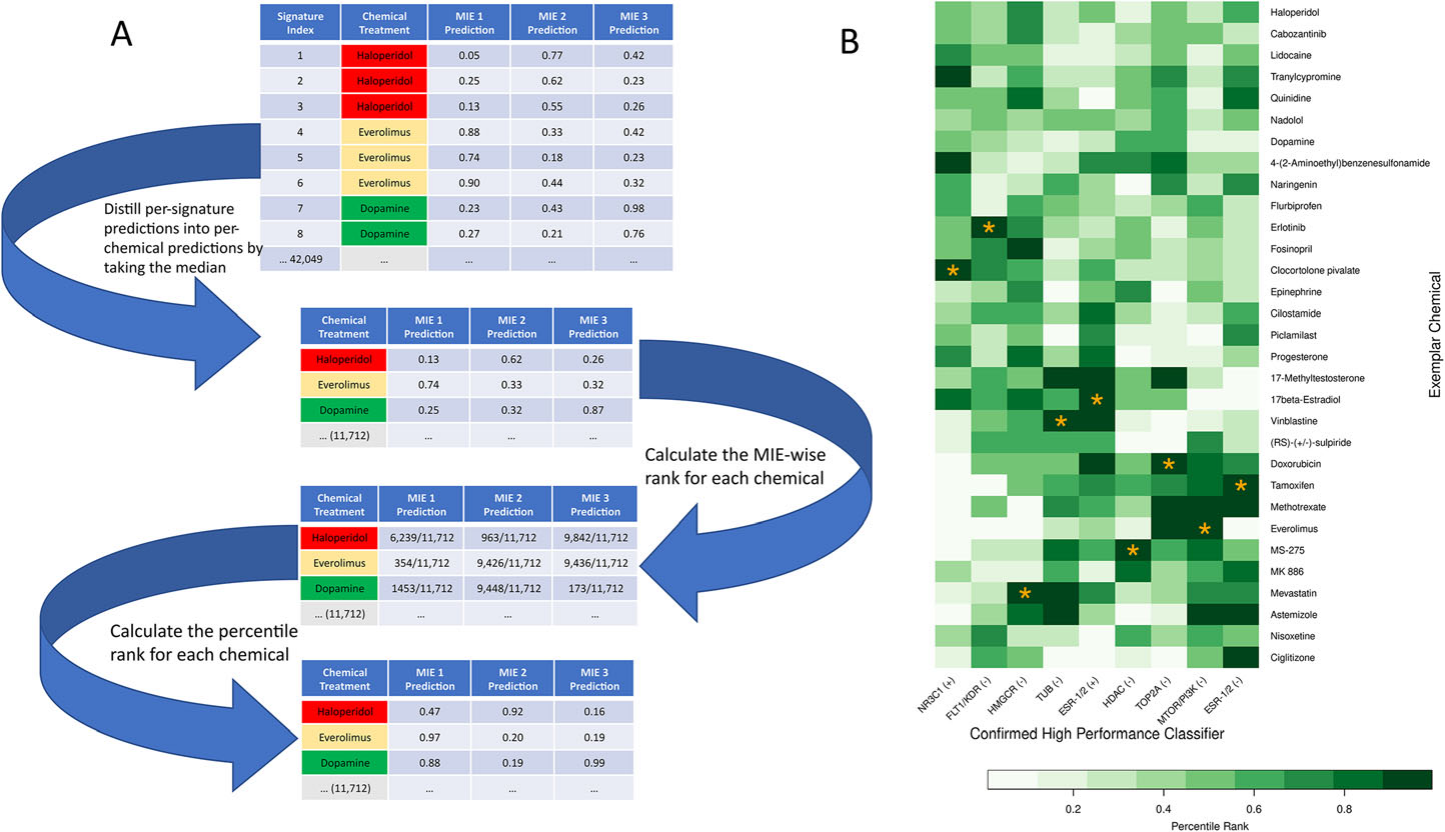
Validation of candidate high-performance classifiers using exemplar chemicals. A) Workflow for converting LINCS profile MIE predictions to within-chemical percentile ranks. B) Heatmap of exemplar chemical percentile ranks for confirmed high performance classifiers. Heatmap columns are organized by classifier. Heatmap rows correspond to training-excluded exemplar chemicals. Cell shading represents the percentile rank for each chemical returned by each classifier. Dark green corresponds to a value of 1 (highest ranked prediction for chemical to activate the MIE) and white corresponds to 0 (lowest ranked prediction for chemical to activate the MIE). Cells marked with a ‘*’ indicate the corresponding exemplar chemical assigned to each MIE based on RefChemDB annotations

In selecting candidate high performance classifiers, models trained with SVM_P were excluded from consideration given the significant differences in internal and holdout accuracy (Fig. 4). Despite this consideration, holdout accuracies for candidate high performance classifiers were significantly lower than internal accuracies (pvalue = 0.018, one tailed paired Wilcoxon test), underscoring the need for additional validation. To achieve this, classifier performance was validated using training-excluded exemplar chemicals. Gene expression profiles from LINCS were imputed into candidate high performance classifiers, generating MIE activation predictions. To summarize exemplar predictions on a per-chemical basis, we took the median of prediction scores for a particular classifier across all LINCS profiles associated with the same chemical. To make exemplar chemical predictions comparable across classifiers, we converted these predictions into percentile ranks (Fig. 6A). Candidate high performance classifiers were then evaluated by the percentile rank of their corresponding exemplar chemical (Table 3). Of the 18 candidate high performance classifiers for which exemplar chemicals were available, 9 classifiers ranked their exemplar chemicals among the top 10% of all LINCS chemicals (bolded rows, Table 3). Candidate models with exemplar chemicals that failed to meet this threshold were excluded from further consideration. We then evaluated the performance of the remaining 9 confirmed high performance classifiers by inspecting the predictions for all exemplar chemicals (Fig. 6B). Erlotinib, Clocortolone pivalate, 17 beta Estradiol, Tamoxifen, and MS-275 showed top-ranking predictions for their respective classifiers (FLT (−), MR3C1 (+), ESR-1/2 (+), ESR-1/2 (−), and HDAC (−)). However, vinblastine, doxorubicin, everolimus, and mevastatin also produced high-ranking predictions for other classifiers associated with MIEs for which they were not annotated.

**Table 3:** Validation Statistics for MCF7 Trained Candidate High Performance Classifiers

MIE Name	Exemplar Chemical	Exemplar Rank	Exemplar Percent Rank	KS Test Chemicals	KS Test *p*-value
ADRA2A (+)	Epinephrine	2826 / 11,666	0.76	2	0.09
ALOX5 (−)	MK 886	4291.5 / 11,672	0.63	3	0.87
AR (+)	17-Methyltestosterone	10,226 / 11,665	0.12	10	0.02
DRD2 (−)	Haloperidol	3042 / 11,623	0.74	8	0.09
**ESR-1/2 (−)**	**Tamoxifen**	**26 / 11,661**	**1.00**	**5**	**0.10**
**ESR-1/2 (+)**	**17beta-Estradiol**	**517 / 11,604**	**0.96**	**22**	**0.03**
**FLT1/KDR (−)**	**Erlotinib**	**1125 / 11,620**	**0.90**	**23**	**0.34**
**HDAC (−)**	**MS-275**	**357 / 11,589**	**0.97**	**13**	**0.00**
**HMGCR (−)**	**Mevastatin**	**922 / 11,672**	**0.92**	**3**	**0.00**
HRH1 (−)	Astemizole	8838 / 11,630	0.24	14	0.61
JAK2 (−)	NA	NA	NA	4	0.34
KCNH2 (−)	Haloperidol	3438 / 11,522	0.70	22	0.01
MAPK14 (−)	NA	NA	NA	13	0.87
MET (−)	Cabozantinib	5357 / 11,626	0.54	3	0.56
**MTOR/PI3K (−)**	**Everolimus**	**3 / 11,572**	**1.00**	**11**	**0.01**
**NR3C1 (+)**	**Clocortolone pivalate**	**322 / 11,639**	**0.97**	**19**	**0.02**
PTGS-1/2 (−)	Flurbiprofen	4782 / 11,556	0.59	20	0.41
SLC22A6 (−)	Methotrexate	8663 / 11,671	0.26	18	0.52
**TOP2A (−)**	**Doxorubicin**	**16 / 11,652**	**1.00**	**5**	**0.02**
**TUB (−)**	**Vinblastine**	**27 / 11,636**	**1.00**	**6**	**0.00**

Bolded entries are the nine confirmed high performance classifiers as determined by an exemplar percent rank ≥0.90

### Confirmed high performance classifiers show high-ranking predictions for MIE-linked chemicals not used in training

To further validate the remaining 9 confirmed high performance classifiers, we leveraged RefChemDB chemical-MIE associations with a moderate support level (support level 3–4). These linkages were previously excluded from training data sets because a previous comparison to in vitro assay results has shown that these associations are more likely to be spurious relative to linkages with a higher support level [16]. On the other hand, we reasoned that even if a subset of chemical-MIE associations are spurious, moderate-support annotations should be enriched for real chemical-MIE relationships. If a sufficient number of such moderate-support annotations are available for a high performance classifier, this enrichment of higher prediction scores should be statistically significant. Importantly, this test involves a set of chemicals that were excluded from the original training data sets.

To test this, we compared prediction ranks for these moderate support chemicals to the background rank distribution using a one-tailed KS test. Of the 9 confirmed high performance classifiers, 8 generated ranked predictions for moderate-support linkages that were significantly higher than the background distribution (Table 3, Supplemental Figure 3). Thus, confirmed high performance classifiers return relatively high predictions for their RefChemDB-associated chemicals not used in classifier training.

### Model performance varies as a function of training data cell line

**Fig. 7:**
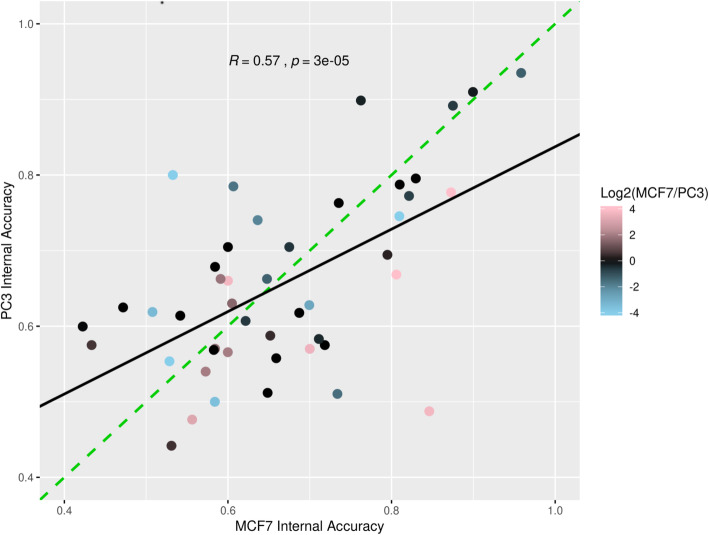
Classifier accuracy across cell types. 46 MIEs are plotted by internal accuracy score for models trained using MCF7 derived data (X axis) and for models trained using PC3 derived data (Y axis). For both cell types, internal accuracy scores are computed based on the SVM Linear (SVM_L) algorithm using landmark genes as the feature type. The dashed identity line is shown in green (slope = 1, intercept = 0). The best fit line is shown in black (slope = 0.55, intercept = 0.29). The correlation coefficient R and corresponding t-test *p*-value are shown at the top of the plot. Points are shaded by the disparity in expression of the associated MIE target gene(s) between cell lines, based on the log2 fold change in NX values from the Human Protein Atlas. Red and blue points correspond to higher relative expression in MCF7 and PC3 cell lines, respectively. For visualization purposes, values greater than 4 and less than − 4 were capped at 4 and − 4, respectively

To explore whether the ability to predict MIEs varies across celluar contexts, a separate series of classifiers was trained on profiles from chemical treatments of the PC3 cell line. Of the 51 MIEs modeled with MCF7-trained data, 46 (91%) had sufficient training data in PC3 cells. To train PC3 classifiers comparable to MCF7-trained counterparts, we used landmark gene features. In order to maximize comparability between classifiers trained in each cell line, we selected SVM_L-based classifiers for comparison. Classifiers trained with SVM_L on MCF7-derived profiles were compared with algorithm and MIE-matched PC3-derived classifiers based on internal accuracies (Fig. 7). MCF7 and PC3-trained classifiers showed significant correlation between accuracies (r = 0.57, pval = 3 × 10^− 5^). Additionally, the classifiers with the highest internal accuracies were the same in both cell types (TUB (−) and MTOR/PI3K (−)). On the other hand, a subset of models showed relatively dissimilar accuracies between cell types. The AR (+) model showed relatively higher internal accuracy when trained on MCF7 data (0.85) compared to training on PC3 data (0.49), while ADRB-1/2 (−) showed relatively higher accuracy when trained on PC3 data (0.80) as opposed to MCF7 (0.53). Because MCF7 cells are known to be estrogen-responsive, we suspected that a greater diversity of estrogen receptor-interacting chemicals may have been assayed in MCF7 cells relative to PC3 cells. Such an imbalance in training data could potentially drive differences in model accuracy between the cell types. However, an inspection of the training data for these classifiers reveals similar numbers of chemicals and profiles were available for ESR-1/2 activation in both cell types (Table 4).

**Table 4:** Classifier Statistics for MIEs Modeled using MCF7 or PC3 Profiles

MIE Name	MCF7 Accuracy	MCF7 MIE Active Profiles	MCF7 MIE Active Chemicals	PC3 Accuracy	PC3 MIE Active Profiles	PC3 MIE Active Chemicals	Accuracy Difference (MCF7 – PC3)	L2FC NX Value
AR (+)	0.85	52	8	0.49	50	8	0.36	3.70
ADRB-1/3 (+)	0.73	51	6	0.51	52	7	0.22	−1.58
ALOX5 (−)	0.72	51	5	0.58	50	6	0.14	0.00
ESR-1/2 (+)	0.81	145	12	0.67	148	12	0.14	5.43
ADRA2A (+)	0.65	58	7	0.51	51	7	0.14	0.00
SCN5A (−)	0.70	100	9	0.57	92	9	0.13	3.58
PDE4-A/B/D (−)	0.71	56	4	0.58	51	5	0.13	−0.35
CYP3A4 (−)	0.66	144	13	0.56	121	12	0.10	0.00
EGFR/ERBB2 (−)	0.79	140	9	0.69	112	9	0.10	0.30
ESR-1/2 (−)	0.87	68	5	0.78	67	5	0.10	5.43
ACE (−)	0.53	57	6	0.44	54	6	0.09	0.49
PDE3A (−)	0.58	67	5	0.50	56	5	0.08	−3.58
SLC22A6 (−)	0.56	55	6	0.48	52	6	0.08	3.17
FLT1/KDR (−)	0.70	122	10	0.63	92	10	0.07	−2.81
CA-1/2 (−)	0.69	90	13	0.62	63	13	0.07	0.00
CYP2D6 (−)	0.65	52	5	0.59	50	5	0.06	0.42
MET (−)	0.81	114	7	0.75	66	7	0.06	−4.60
HDAC (−)	0.82	174	10	0.77	153	10	0.05	−0.71
KCNH2 (−)	0.60	369	34	0.57	304	33	0.03	2.00
PDGFRB (−)	0.83	66	6	0.80	54	6	0.03	0.00
CYP19A1 (−)	0.57	70	7	0.54	66	7	0.03	2.00
TUB (−)	0.96	104	8	0.94	66	8	0.02	−1.29
KIT (−)	0.81	88	5	0.79	76	5	0.02	0.00
PTGS-1/2 (−)	0.62	247	28	0.61	227	28	0.01	−0.58
HTR2A (−)	0.58	67	8	0.57	64	7	0.01	0.00
DRD2 (−)	0.58	118	14	0.57	114	15	0.01	1.58
MTOR/PI3K (−)	0.90	204	12	0.91	146	12	−0.01	−0.23
SCN-1/2-A (−)	0.65	51	5	0.66	50	5	−0.01	−1.32
TOP2A (−)	0.88	75	7	0.89	58	6	−0.02	0.61
HRH1 (−)	0.53	110	14	0.55	94	14	−0.02	−5.04
DRD2 (+)	0.61	125	15	0.63	115	15	−0.03	1.58
FLT3 (−)	0.74	84	5	0.76	60	5	−0.03	0.00
MAO-A/B (−)	0.68	75	11	0.70	69	10	−0.03	−0.49
AR (−)	0.60	56	5	0.66	54	5	−0.06	3.70
CA-9/12 (−)	0.59	76	10	0.66	50	10	−0.07	1.82
ABCB1 (−)	0.54	283	25	0.61	236	25	−0.07	0.00
SLC6A-2/3/4 (−)	0.58	192	18	0.68	178	18	−0.09	0.00
PPAR-A/D/G (+)	0.64	137	16	0.74	129	16	−0.10	−2.00
NR1I2 (+)	0.60	50	6	0.70	67	6	−0.10	0.00
ADRB-1/2 (−)	0.51	67	8	0.62	58	8	−0.11	−3.39
NR3C1 (+)	0.76	100	10	0.90	92	10	−0.14	−0.28
DHFR/TYMS (−)	0.43	56	6	0.58	54	6	−0.14	0.57
ADRA1A (+)	0.47	67	10	0.62	63	10	−0.15	0.00
APP (−)	0.42	71	5	0.60	70	5	−0.18	−0.04
SLC22A-1/2 (−)	0.61	63	7	0.78	55	6	−0.18	−1.70
ADRB2 (+)	0.53	55	9	0.80	50	9	−0.27	−6.73

An alternative explanation for these differences in classifier accuracies between MIE-matched models is an underlying difference in expression of the relevant target genes between cell lines. To investigate this further, we downloaded normalized expression (NX) values from the Human Protein Atlas v20 [33] for MIE target genes in MCF7 and PC3 cell lines. These values represent transcripts per million derived from RNA-seq profiling that were further normalized with a trimmed mean approach, pareto scaling, and batch correction, and are specifically intended for comparison of relative mRNA levels of individual genes across diverse cell lines. For MIEs that were associated with multiple genes (discussed in section 2.1), a representative NX value was calculated by taking the median NX value from the set of gene targets contained in the MIE. Based on these data, we hypothesize that the increased performance of the AR (+) classifier trained on MCF7 data is likely a result of higher expression of AR in MCF7 cells (NX: 1.2) compared to PC3 cells (NX: 0.0). Conversely, we found that ADRB2 mRNA was not detectable in MCF7 cells but shows strong expression in PC3 (NX: 10.5), which corresponds to the increased accuracy of the ADRB-1/2 (−) classifier. To explore this relationship systematically, we calculated the log2 fold-change in NX values between the two cell lines for all MIE target genes (MCF7/PC3, adding a pseudo count of 0.1 to both values, see Fig. 7 and last column in Table 4). In particular, we found that MIE classifiers corresponding to target genes with substantially higher expression in MCF7 cells tended to have better overall performance when trained on MCF7 data. Thus, differences in classifier performance across cell type correspond in part to differences in the expression of the target proteins underlying each MIE, but are not fully explained by simple analysis of the target gene mRNA expression.

## Discussion

In the current study, we trained binary classifiers to predict MIEs from gene expression profiles and optimized both input feature type and classification algorithm. By integrating LINCS and RefChemDB in a ML framework for MIE prediction, our analysis has the following primary findings: 1) We identified landmark genes as an optimal feature type for MIE prediction based on internal accuracies, 2) Of the 51 MIEs modeled, 9 MIEs generated classifiers that performed significantly better than null models and that were further validated using training-excluded chemicals and profiles, and 3) Classifier accuracy varied based on the cell line from which training data were derived, and may be associated with differences in baseline expression of MIE-linked genes.

Previous studies of drug-use category prediction showed that classifiers trained on pathway scores outperformed analogous models trained on individual gene features [13], prompting our evaluation of pathway score-based classifiers. Surprisingly, we found that models trained on pathway features underperformed relative to models trained on landmark genes. This may be attributable to differences in the level of granularity modeled in each study. Our work attempts to predict chemical bioactivity at the level of specific molecular targets, as contrasted with previous work [13], which trained classifiers using labels linked to broader therapeutic use categories.

Previous ML-based investigations incorporating LINCS L1000 data have shown that deep-learning-based approaches yielded superior accuracy in predicting general chemical use categories relative to SVM-based classifiers [13]. We were therefore surprised that SVM classifiers out-competed multilayer perceptron in our analysis. One possible explanation is that unlike previous work [13], in which a single multi-classifier was trained to predict broader chemical use-case labels, our approach involved training a separate binary classifier for each MIE, a task for which SVMs are generally well-suited [26]. Another possible contributor to the relatively low accuracy of our MLP-based classifiers is that the default hyperparameters for this training algorithm (as encoded in the R library *caret*) are limited to a single hidden layer due to compute time constraints. However, the training and validation framework presented in the current work can be used to test additional models and hyperparameter combinations in the future.

Confirmed high performance classifiers assigned relatively high prediction scores to the corresponding exemplar chemicals, which were completely excluded from the training data. However, some classifiers assigned high ranking prediction scores to chemicals for which they were not annotated. For example, Everolimus received a high ranking prediction for MTOR/PI3K (−), to which it is linked through RefChemDB annotations, but was also a high-ranking prediction for TOP2A (−), for which it is not annotated. These MIEs are related in that induction of either can be linked to decreased cell proliferation. MTOR (Mechanistic Target of Rapamycin) and a downstream pathway that includes Phosphoinositide 3-kinase (PI3K) is a major regulator of cell growth and proliferation, while TOP2A (Topoisomerase 2-alpha), is a nuclear enzyme that alters topology of DNA to facilitate replication. The high ranking prediction of Everolimus for TOP2A (−) may therefore result from the convergence of distinct MIEs onto the same key events through shared molecular consequences. While Everolimus is known to inhibit MTOR but not TOP2A, the inhibition of either of these targets is known to induce apoptosis [34, 35]. Similar cross-talk between antiproliferative pathways in MCF7 cells (MTOR/PI3K (−), TOP2A (−), ESR-1/2 (−)) likely also explains the high-ranking predictions for Methotrexate (Fig. 6B), a chemotherapeutic that acts via inhibition of dihydrofolate reductase [36]. These off-target positive predictions may therefore reflect a limitation of the current methodology. However, we emphasize that the current methods were developed to predict potential hazards in tier-1 high-throughput screening efforts [3], and in that framework would require additional validation with orthogonal assays to confirm relationships between chemicals and their respective MIEs. Therefore, the primary consideration in terms of predictive performance is the sensitivity of the confirmed high performance classifiers, as demonstrated by their ability to recover training-excluded exemplar chemicals among the top 10% of predictions.

An additional limitation of the current work is sparse data on which to train classifiers. Previous ML-based investigations have leveraged more general labels capturing overall toxicity or chemical use categories to train classifiers using expression profiles associated with hundreds of chemicals. In contrast, models produced by the current work are trained using as few as 5 chemicals associated with much more specific biological activities, such as AR inhibition. One reason for this sparseness in the training data is that currently there are relatively few chemicals associated with certain MIEs with high levels of literature support. RefChemDB is the product of an automated curation process and therefore contains some spurious chemical-target associations. To ensure the validity of our training data, we limited MIE-active chemicals to those with a support level corresponding to at least 5 separate sources of evidence for the association. It is likely that a subset of chemical-MIE annotations with lower support levels are valid and could therefore be added to the available training data as additional evidence becomes available. A second contributor to the sparseness of the training data is limited overlap between chemicals annotated in RefChemDB, and chemicals surveyed in LINCS. Of the 1181 chemicals annotated in RefChemDB with a support level ≥ 5, only 765 (65%) have available data in LINCS.

We addressed the sparseness of the data by first clustering similar targets into a single MIE, such as HDAC1 (−) and HDAC2 (−), using a data-driven strategy based on overlapping chemical associations in RefChemDB. This strategy generated MIEs that can easily be associated with AOPs, without unnecessarily distinguishing between gene family members for which there are a limited number of chemicals that display family member specific affinity. We also increased the size of our training data sets by leveraging multiple examples of each chemical treatment in the LINCS data, including those tested at multiple concentrations and exposure durations. We also performed an empirical significance test using null models matched to the number of chemicals and profiles used to train each MIE model. This test should identify cases where the model accuracy is likely to reflect spurious commonalities between profiles for a small number of chemicals, rather than the true transcriptional signature of the MIE.

Another limitation of the current methodology was the data-driven selection of exemplar chemicals. This selection paradigm considered MIE-chemical support level, frequency of annotation across MIEs, and minimal training data set volume to identify suitable exemplar chemicals (see section 2.8). We used this data-driven approach for exemplar selection to remove our own bias of chemical-MIE linkages from the analysis. However, performing this selection without expert knowledge of metabolism and molecular crosstalk may have resulted in less than ideal exemplar chemicals. For example, our strictly data-driven method selected 17-Methyltestosterone as the exemplar for AR (+). AR (+) passed empirical significance testing (*p*-value = 0.03) but failed to generate a high-ranking prediction for 17-Methyltestosterone (87.66 percentile). On the other hand, 17-Methyltestosterone did return high-ranking predictions for TUB (−), ESR-1/2 (+), and TOP2A (−), none of which are linked to the chemical in RefChemDB. Consideration of the literature reveals 17-Methyltestosterone is estrogenic due to the ease of its conversion to estrogen by aromatase in MCF7 cells, which may explain the high ranking prediction for ESR-1/2 (+) [37].

To explore if MIE predictions differed as a function of the cellular context of training data, we compared classifiers trained on MCF7-derived data with models trained on PC3-derived data. Of the models with sufficient gene expression profiles in LINCS for training in both cell types, a subset, such as TUB (−), showed relatively high and comparable internal accuracy across cell lines. For MIEs modeled by classifiers that demonstrate comparable performance across cell lines, it may be advantageous to attempt to improve classifier accuracy further by training classifiers with an expanded set of training data derived from multiple cell lines in LINCS.

While some classifiers showed relatively similar results across both cell lines, a subset of MIE classifiers showed markedly different internal accuracies as a function of the cell line on which they were trained (Table 4). This disparity in performance could not be explained by differences in the volume of LINCS L1000 data available for model training in each cell type, but was partially associated with differences in baseline expression of MIE target genes in each cell line. However, some MIEs, such as NR3C1 (+) showed a disparity in classifier performance despite similar baseline mRNA expression levels reported in Human Protein Atlas (MCF7 NX: 3.6; PC3 NX: 4.4) It is also possible that such differences in classifier performance are attributable to cell line specific differences in the expression of cofactors and other signaling molecules that enhance or repress the responsiveness to a particular MIE signal, though this is not explored in the current study.

While the relative responsiveness to estrogen receptor modulators in MCF7 cells is well studied [38], the relative suitability for screening various other MIEs across different cellular contexts has not been broadly explored. Thus, in addition to predicting chemical bioactivity, the methods presented here may also provide utility in selecting which cell lines are the most informative for screening candidate compounds for activation of specific MIEs based on the relative performance of classifiers trained on data from different cell types.

## Conclusions

The current study used a binary classification approach to predict MIEs induced by chemical exposure based on gene expression data. We explored a variety of modeling parameters including input feature type, classification algorithm, and cellular context of training data. Candidate high performance classifiers were identified with empirical significance testing, and further validated using training-excluded exemplar reference chemicals.

Systematic comparison of models generated with MCF7 and PC3 derived profiles identified MIEs that were modeled with markedly different accuracies in each cell type, emphasizing the importance of cellular context in model training. As demonstrated by training-excluded exemplar chemical predictions, we propose that a subset of classifiers offer utility in predicting MIEs from L1000 gene expression data. Methods developed herein could be integrated into EPA’s current tiered testing paradigm to prioritize chemicals for further study within a framework of new approach methodologies [3]. Future work will involve the evaluation of model performance on an expanded set of L1000 gene expression profiles, as well as an investigation of the extensibility of LINCS L1000 trained classifiers in successfully predicting MIES using gene expression data produced on other HTTr platforms.

## Supplementary Information

**Additional file 1: Supplemental Fig. 1.** Distribution of LINCS gene expression profiles across cell lines. Each slice of the pie chart captures the percent of all LINCS phase I and II chemical perturbagen gene expression profiles that are derived from the indicated cell types. 76 Cell lines associated with < 5% of total profiles each were combined into the “all other cell lines” group.

**Additional file 2: Supplemental Fig. 2.** Original and null model accuracies as a function of the number of training chemicals. A) Linear regression of internal accuracy (Y axis) for each MIE using each of the six classification algorithms as a function of the number of chemicals included in model training for MCF7-derived landmark gene classifiers (X axis). B) Linear regression of mean internal accuracy from 500 corresponding null classifiers (Y axis) for each MIE is as a function of the number of chemicals used in training (X axis).

**Additional file 3: Supplemental Fig. 3.** Enrichment of high-ranking prediction scores among training excluded chemicals for confirmed high performance classifiers. For each of 9 confirmed high performance classifier, an Empirical Cumulative Distribution Function (ECDF) is plotted for LINCS chemicals ranked by median prediction. Solid red lines correspond to the ECDFs for MIE-associated chemicals with a support level of 3 or 4 in RefChemDB. Blue lines correspond to the ECDFs for all chemicals tested in MCF7 cells. Red dots and dashed lines indicate the greatest difference between the ECDF functions, which is used as the test statistic for the Kolmogorov-Smirnov (KS) test *p*-values shown above each plot.

## Data Availability

Modeling data and analysis scripts generated during the current study are available in the github repository: https://github.com/USEPA/CompTox-MIEML. RefChemDB is available for download as supplemental material from its original publication (PMID: 30570668). LINCS gene expression data are publicly available and accessible through the gene expression omnibus (GSE92742 and GSE70138) at https://www.ncbi.nlm.nih.gov/geo/ .

## Abbreviations

MIEs: Molecular Initiating Events
LINCS: Library of Integrated Cellular Signatures
NAMs: New Approach Methodologies
HTTr: High Throughput Transcriptomics
AOP: Adverse Outcome Pathway
ML: Machine Learning
SVM: Support Vector Machine
EPA: Environmental Protection Agency
KNN: K-Nearest Neighbor
MLP: Multilayer Perceptron
NB: Naïve Bayes
DTXSID: DSSTox Substance Identifier
ssGSEA: single-sample Gene Set Enrichment Analysis
NX: Normalized Expression
ECDF: Empirical Cumulative Distribution Function
KS: Kolmogorov-Smirnov
