# Supplementary figures and images for: Predicting molecular initiating events using chemical target annotations and gene expression
Source: BioData Min. 2022 Mar 4;15:7. doi: 10.1186/s13040-022-00292-z (PMC8895536; doi:10.1186/s13040-022-00292-z)

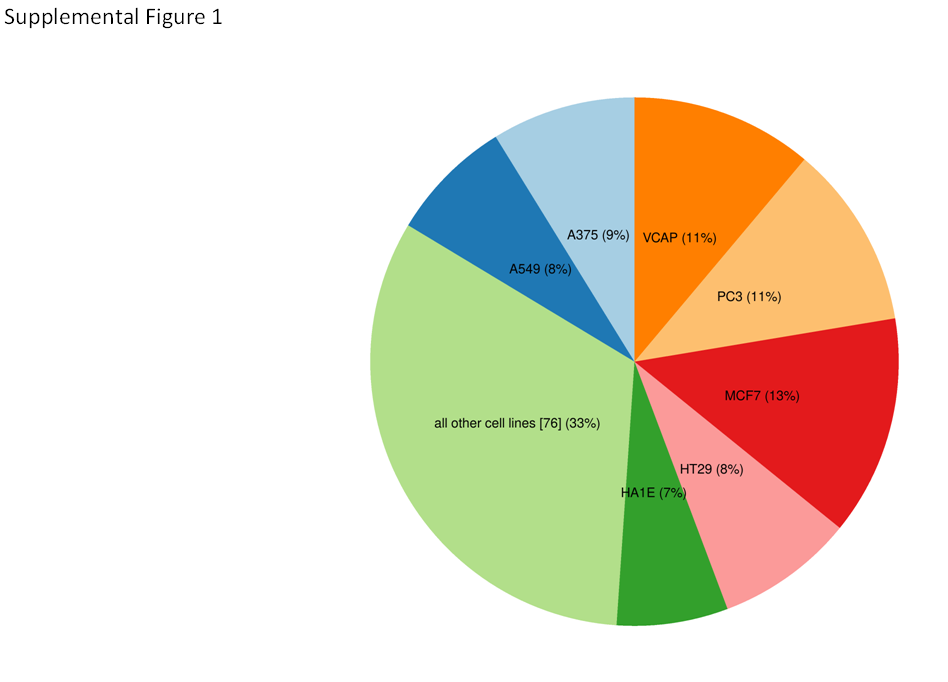

Supplement: Supplementary file 1 — Additional file 1: Supplemental Fig. 1. Distribution of LINCS gene expression profiles across cell lines. Each slice of the pie chart captures the percent of all LINCS phase I and II chemical perturbagen gene expression profiles that are derived from the indicated cell types. 76 Cell lines associated with < 5% of total profiles each were combined into the “all other cell lines” group. [file 13040_2022_292_MOESM1_ESM.png]

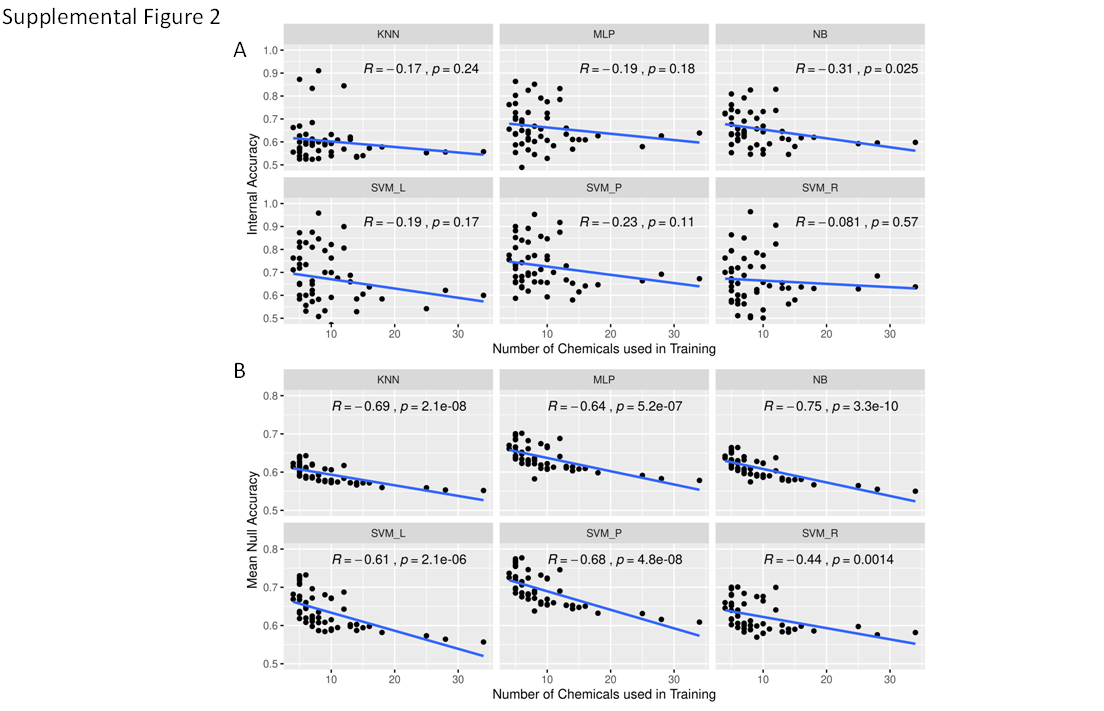

Supplement: Supplementary file 2 — Additional file 2: Supplemental Fig. 2. Original and null model accuracies as a function of the number of training chemicals. A) Linear regression of internal accuracy (Y axis) for each MIE using each of the six classification algorithms as a function of the number of chemicals included in model training for MCF7-derived landmark gene classifiers (X axis). B) Linear regression of mean internal accuracy from 500 corresponding null classifiers (Y axis) for each MIE is as a function of the number of chemicals used in training (X axis). [file 13040_2022_292_MOESM2_ESM.png]

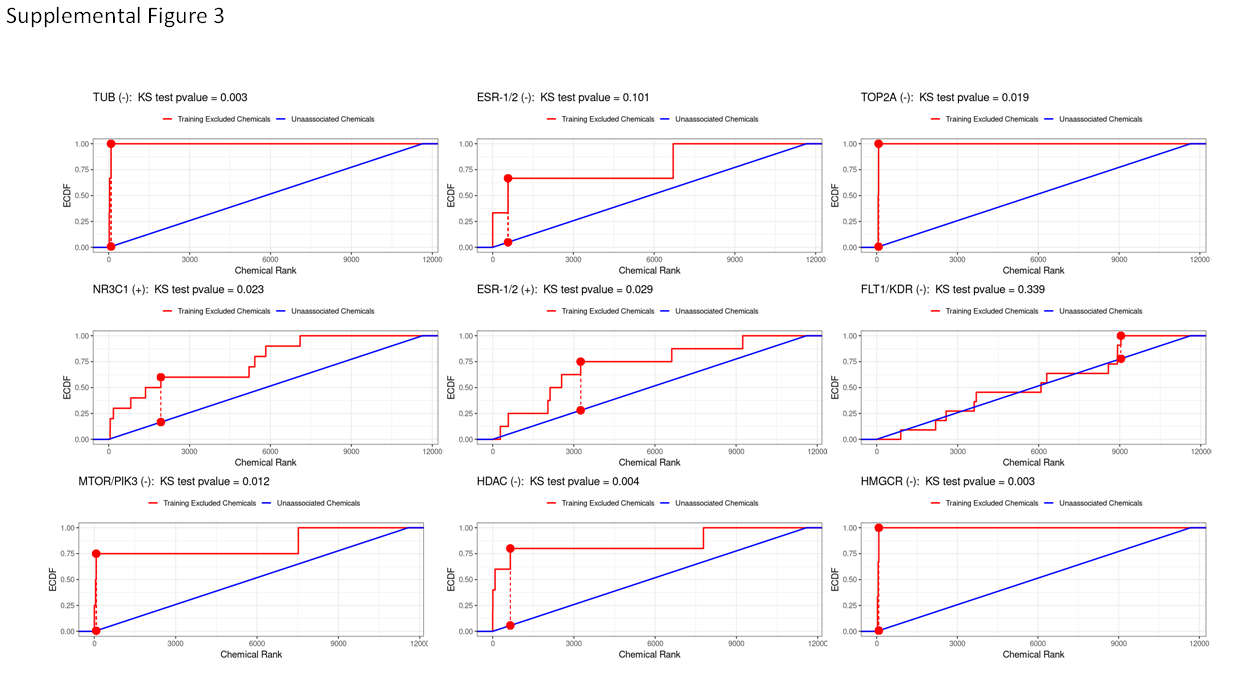

Supplement: Supplementary file 3 — Additional file 3: Supplemental Fig. 3. Enrichment of high-ranking prediction scores among training excluded chemicals for confirmed high performance classifiers. For each of 9 confirmed high performance classifier, an Empirical Cumulative Distribution Function (ECDF) is plotted for LINCS chemicals ranked by median prediction. Solid red lines correspond to the ECDFs for MIE-associated chemicals with a support level of 3 or 4 in RefChemDB. Blue lines correspond to the ECDFs for all chemicals tested in MCF7 cells. Red dots and dashed lines indicate the greatest difference between the ECDF functions, which is used as the test statistic for the Kolmogorov-Smirnov (KS) test p-values shown above each plot. [file 13040_2022_292_MOESM3_ESM.png]
